# Differential effects of dietary fibres on colonic barrier function in elderly individuals with gastrointestinal symptoms

**DOI:** 10.1038/s41598-018-31492-5

**Published:** 2018-09-07

**Authors:** J. P. Ganda Mall, L. Löfvendahl, C. M. Lindqvist, R. J. Brummer, Å. V. Keita, I. Schoultz

**Affiliations:** 1 Nutrition-Gut-Brain Interactions Research Centre, School of Medical Sciences, Faculty of Medicine and Health, Örebro University, Örebro, Sweden; 2 Nutrition and physical activity research centre, School of Medical Sciences, Faculty of Medicine and Health, Örebro University, Örebro, Sweden; 3 Department of Clinical and Experimental Medicine, Linköping University, Linköping, Sweden

## Abstract

Gastrointestinal problems are common in elderly and often associated with psychological distress and increased levels of corticotrophin-releasing hormone, a hormone known to cause mast cell (MC) degranulation and perturbed intestinal barrier function. We investigated if dietary fibres (non-digestible polysaccharides [NPS]) could attenuate MC-induced colonic hyperpermeability in elderly with gastrointestinal (GI) symptoms. Colonic biopsies from elderly with diarrhoea and/or constipation (n = 18) and healthy controls (n = 19) were mounted in Ussing chambers and pre-stimulated with a yeast-derived beta (β)-glucan (0.5 mg/ml) or wheat-derived arabinoxylan (0.1 mg/ml) before the addition of the MC-degranulator Compound (C) 48/80 (10 ng/ml). Permeability markers were compared pre and post exposure to C48/80 in both groups and revealed higher baseline permeability in elderly with GI symptoms. β-glucan significantly attenuated C48/80-induced hyperpermeability in elderly with GI symptoms but not in healthy controls. Arabinoxylan reduced MC-induced paracellular and transcellular hyperpermeability across the colonic mucosa of healthy controls, but did only attenuate transcellular permeability in elderly with GI symptoms. Our novel findings indicate that NPS affect the intestinal barrier differently depending on the presence of GI symptoms and could be important in the treatment of moderate constipation and/or diarrhoea in elderly.

## Introduction

The prevalence of gastrointestinal (GI) symptoms, such as diarrhoea and constipation, are widespread in the ageing population^1,2^. Nearly 50% of elderly above the age of 55 year’s experience GI symptoms^3,4^ and in nursing homes, the prevalence increases to 70%^5^. Life satisfaction is strongly linked to a well-functioning GI tract^6,7^ and diseases of the digestive system are known to lead to a higher symptom burden affecting the overall health status negatively in the elderly^8^. Previous studies have reported a stronger level of anxiety and depression in elderly with GI symptoms^9,10^. Conditions of psychological distress are common among the elderly population^11–13^ and correlates to increased levels of the stress hormone corticotrophin-releasing hormone (CRH)^14–16^. Increased levels of CRH have been shown to contribute to a dysregulated intestinal barrier function^17^ by interacting directly with mast cells (MCs) and triggering their degranulation. This leads to increased paracellular and transcellular permeability^17^ of foreign substances across the intestinal mucosa, a hallmark of several GI diseases^18–20^ and recently found associated to mental disorders such as depression/anxiety^21,22^ and autism spectrum disorder^23^. Thus, MCs represents a potential link between psychological distress and a disturbed intestinal barrier function which might be one of the mechanisms behind the increased GI problems in the elderly population. Recently, we showed that elderly individuals self-reporting moderate GI symptoms also suffered from psychological distress and displayed signs of a perturbed intestinal barrier function^24^.

Prebiotic dietary fibres, such as non-digestible polysaccharides (NPS), are fermented by the gut microbiota. The fermentation of NPS initiates the proliferation of specific health beneficial bacteria *in situ* and the process also generates short-chain fatty acids (SCFA), such as butyrate^25–27^. Recently, we showed that a yeast-derived beta (β)-glucan was able to interact dicrectly with MCs and reduce hyperpermeability due to MC degranulation in ileal specimens from patients with Crohn’s disease (CD), mounted in Ussing chambers^28^. Given the close relationship between psychological distress and GI symptoms we hypothesised that NPS stimulation could strengthen the colonic barrier function and attenuate MC-induced hyperpermeability. Here we investigate the effect of the NPS arabinoxylan (wheat) and yeast-derived β-glucan (*Saccharomyces cerevisiae*) to counteract MC-induced hyperpermeability across colonic mucosa in biopsies from elderly individuals suffering from constipation and/or diarrhoea.

## Results

### Baseline characteristics

Comorbidities and medical use were reported in the case report forms (CRF) and are presented in Table 1. The usage of cardiovascular drugs (antihypertensive drugs, anti-coagulants, among others) was most prominent in the elderly cohort (40%) followed by gut regulating drugs (30%). The frequency of smoking was low and only one participant in each study group smoked. The food frequency questionnaire (FFQ) further showed that 19 participants of the 20 elderly with GI symptoms had an inadequate dietary fibre intake, with a median of 64.3% (interquartile range [IQR] 53.3–80.7%) of the Nordic Nutrition Recommendations (NNR). The BMI was found not to differ significantly between the two populations. There was a considerably higher proportion of females in the two study groups, with 90% female participants in the elderly cohort and 65% in the healthy control group. Moreover, it is important to point out that there was a significant difference in age between the two study populations, p < 0.001, Table 1.

**Table 1:** Demographic data showcasing the baseline characteristics of the two study populations.

	Elderly with gastrointestinal symptoms	Healthy adults
Gender	**n** = **18**	**n** = **23**
*Female, n (%)*	16 (90%)	15 (65%)
*Male, n (%)*	2 (10%)	8 (35%)
Age, mean ± std	72.4 ± 3.9	24.8 ± 3.0
BMI, median (IQR)^a^	23.9 (22.0–30.5)	22.1 (21.5–24.0)
Smokers, n (%)	1 (5%)	1 (4.7%)
Medications	%	%
*Cardiovascular drugs* ^b^	40.0	0
*Gut regulating drugs* ^c^	30.0	0
*NSAIDs* ^d^	0	0
*Others* ^e^	50.0	8.7
*Polypharmacy* ^f^	15.0	0

^a^IQR – Interquartile range.

^b^Cardiovascular drugs: antihypertensive medications, anti-coagulants, statins.

^c^Gut regulating drugs: probiotics, fibres, laxatives, proton pump inhibitors.

^d^NSAIDs – Non-steroid anti-inflammatory drugs.

^e^Others – Thyroid drugs, sleeping pills, cough medicine, hormones, anti-depressant (10%).

^f^Polypharmacy – 5 or more drugs.

### No difference in colonic permeability between elderly with no GI symptoms and young adults

The influence of age on intestinal permeability was further assessed using a non-invasive multi sugar test. A non-invasive methodology was chosen to allow for the recruitment of a sample size of elderly reporting no GI symptoms large enough for assessment of intestinal permeability. The invasive methodology required to obtain colonic biopsies for Ussing chamber experiments limited the recruitment process and a sufficient number of healthy elderly could not be enrolled. The colonic permeability was assessed in elderly with no GI symptoms and a young healthy control group using a non-invasive multi sugar test. All demographic data over gender distribution; age, BMI, medications and GI symptoms can be viewed in Supplementary Table S1. The multi-sugar test quantifies the 24 h urinary excretion of the ingested sugars sucralose (S) and erythritol (E), where the calculated S/E ratio reflects colonic permeability. The multi-sugar test revealed no difference in basal colonic permeability, as reflected by the S/E ratio (median [IQR]), between younger healthy individuals (0.024 [0.019–0.031]) and elderly individuals reporting no GI symptoms (0.025 [0.017–0.034]).

### Electrophysiological changes

Potential difference (PD), transepithelial resistance (TER) and short-circuit current (Isc) were monitored over time and plotted for the time points 0, 30, 60 and 90 min. The PD of biopsies from both elderly with GI symptoms and healthy adults were confirmed to be within viable range (≤−0.5 mV) for the entire duration of experiments. Changes in TER and Isc values over time for all different treatment conditions are shown in Table 2 and Supplementary Table S3, respectively, describing the electrophysiological state of the biopsies during the experiments. Biopsies stimulated with both yeast-derived β-glucan and Compound (C) 48/80 from both study populations displayed a significantly higher TER over time (mean of data from 30–90 min time points) compared to biopsies stimulated with C48/80 only (Table 2), p < 0.05. No other significant differences were observed for TER or Isc in either study population.

**Table 2:** Transepithelial resistance (TER) values (mean ± SD) with 30 min intervals normalised to each participant’s respective 0 min value presented as percentage.

TER	0 min	30 min	60 min	90 min	*Baseline corrected mean over time (30–90 min)*
Healthy controls (n = 21)					
Vehicle	100.0	91.3 ± 5.9	87.4 ± 6.1	83.6 ± 6.6	*87.4* ± *5.9*
C48/80	100.0	90.0 ± 4.0	86.6 ± 4.5	84.0 ± 5.4	*86.9* ± *4.4*
C48/80 + β-glucan *(n* = *13)*	100.0	94.1 ± 5.9	90.2 ± 6.5	87.5 ± 7.4	*90.6* ± 6.5*
C48/80 + AX	100.0	89.5 ± 3.5	86.0 ± 3.9	83.9 ± 4.0	*86.5* ± *3.7*
β-glucan *(n* = *13)*	100.0	93.4 ± 5.2	89.5 ± 5.3	86.7 ± 5.8	*89.9* ± *5.3*
AX	100.0	91.1 ± 4.0	87.6 ± 3.9	86.6 ± 5.2	*88.4* ± *4.2*
Older adults with gastro-intestinal symptoms (n = 16)					
Vehicle	100.0	91.4 ± 6.1	88.1 ± 6.4	85.1 ± 6.6	*88.2* ± *6.3*
C48/80	100.0	90.2 ± 3.6	87.5 ± 3.9	85.1 ± 4.5	*87.6* ± *3.9*
C48/80 + β-glucan	100.0	94.3 ± 6.9	91.5 ± 7.0	88.8 ± 6.9	*91.5* ± 6.9*
C48/80 + AX	100.0	90.4 ± 3.5	88.0 ± 3.5	86.8 ± 4.6	*88.4* ± *3.7*
β-glucan	100.0	92.1 ± 4.8	89.2 ± 5.5	87.3 ± 6.3	*89.5* ± *5.4*
AX	100.0	91.9 ± 4.7	89.8 ± 5.1	89.4 ± 6.3	*93.6* ± *14.9*

*p < 0.05 - statistically significant compared to C48/80 using the paired t-test.

Arabinoxylan (AX), Compound 48/80 (C48/80).

### Yeast-derived β-glucan attenuates MC-induced hyperpermeability in elderly with GI symptoms

**Figure 1:**
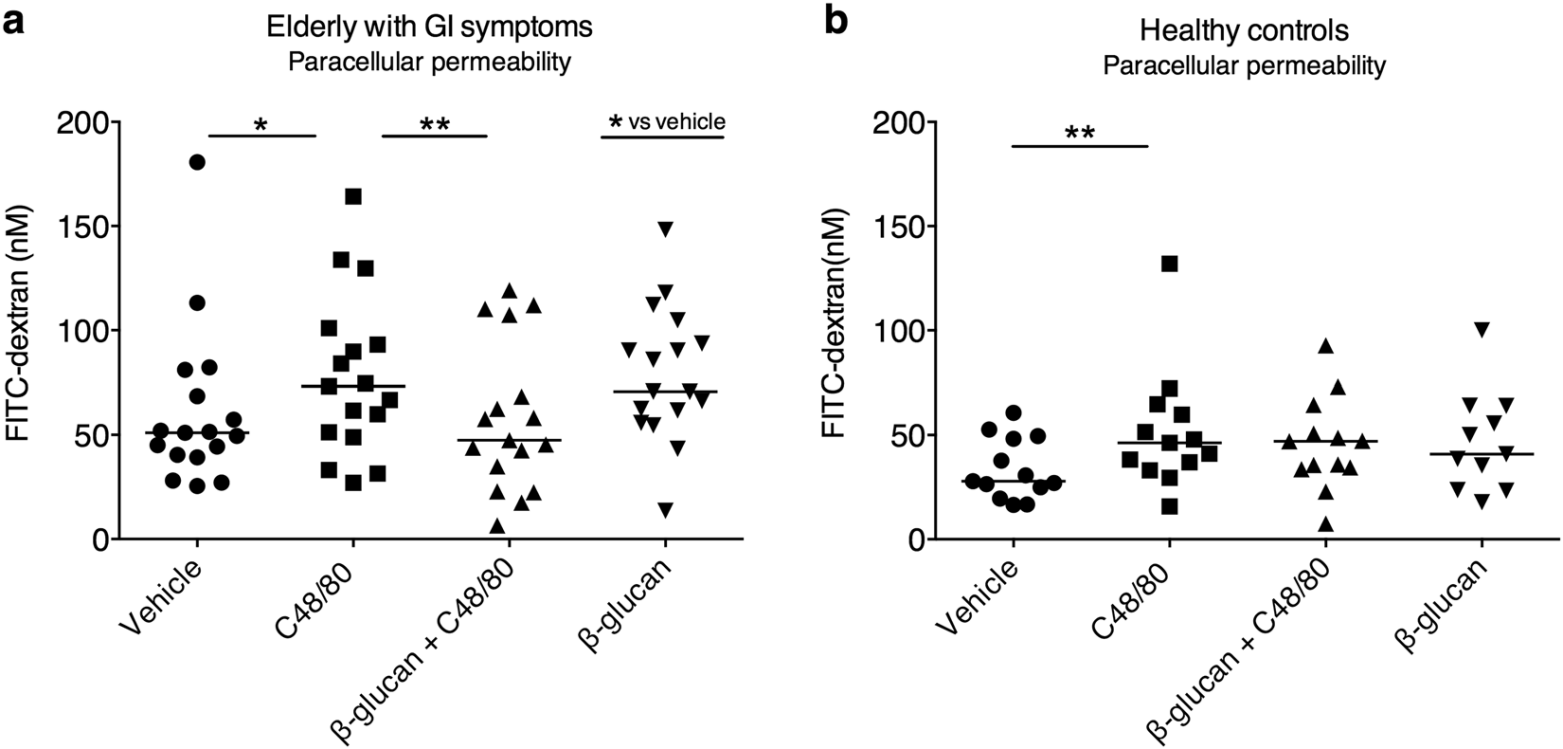
Effects of yeast-derived β-glucan on colonic paracellular permeability in biopsies mounted in Ussing chambers. Stimulation with Compound (C) 48/80 significantly increased the paracellular permeability compared to vehicle in both elderly with gastrointestinal (GI) symptoms (**a**, n = 17) and healthy controls (**b**, n = 13). Co-stimulation with β-glucan attenuated C48/80 induced paracellular hyperpermeability in elderly with GI symptoms but not healthy controls. Stimulation with β-glucan alone displayed significantly increased paracellular permeability compared to vehicle in elderly with GI symptoms but not healthy controls (n = 11). Data (∆90—0 min) is presented as a line intersecting the median and each dot represents one participant, *p < 0.05, **p < 0.01, ns = non-significant. One older adult had to be excluded from FITC-analysis due to technical problems, hence total number 17 instead of 18.

**Figure 2:**
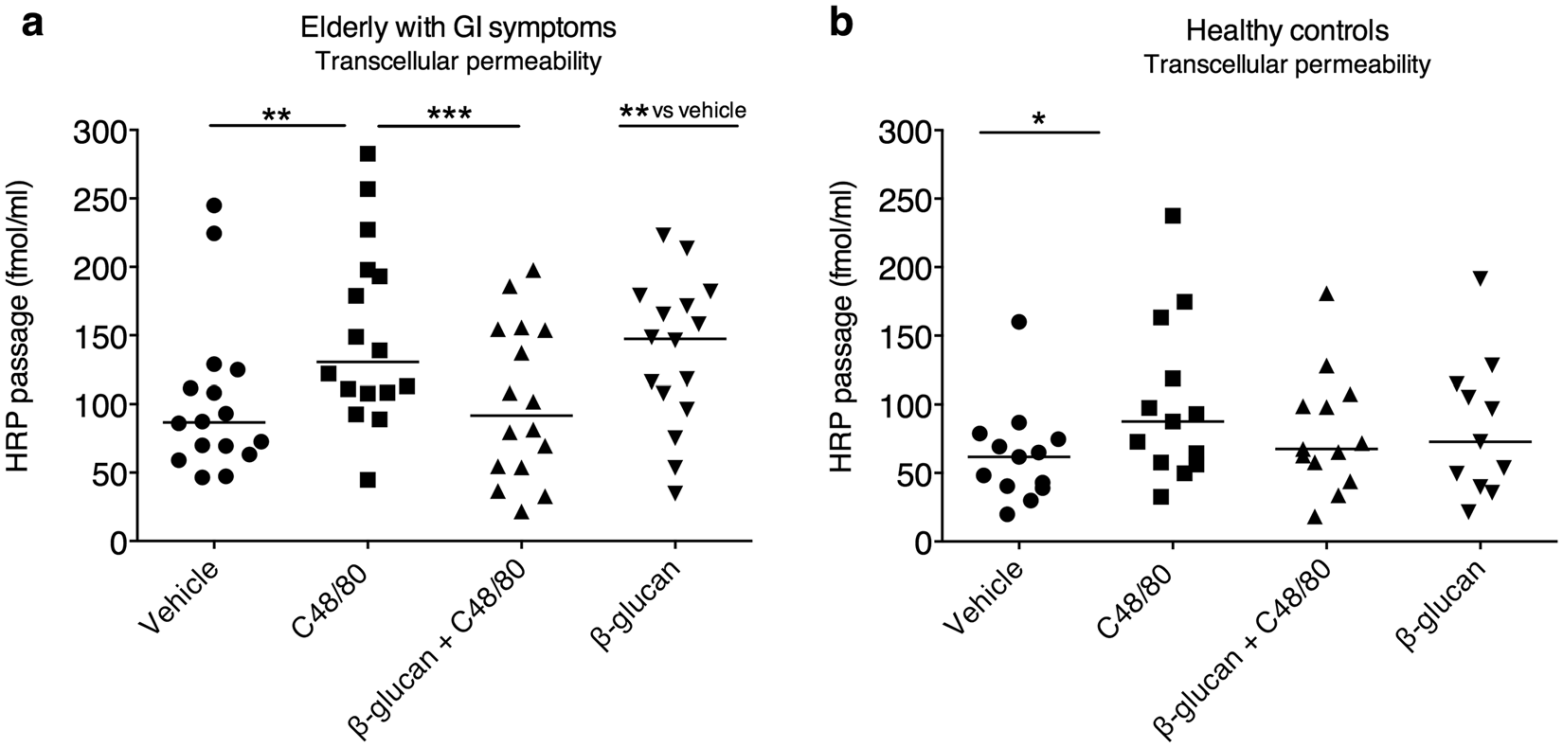
Effects of yeast-derived β-glucan on colonic transcellular permeability in biopsies mounted in Ussing chambers. Stimulation with Compound (C) 48/80 significantly increased the transcellular permeability compared to vehicle in both elderly with gastrointestinal (GI) symptoms (**a**, n = 16) and healthy controls (**b**, n = 13). Co-stimulation with β-glucan attenuated C48/80 induced transcellular hyperpermeability in elderly with GI symptoms but not healthy controls. Stimulation with β-glucan only displayed significantly increased transcellular permeability compared to vehicle in elderly with GI symptoms but not healthy controls (n = 11). Data (∆90—0 min) is presented as a line intersecting the median and each dot represents one participant, *p < 0.05, **p < 0.01, ***p < 0.001, ns = non-significant. Two elderly had to be excluded from horseradish peroxidase (HRP)-analysis due to technical problems, hence total number 16 instead of 18.

Colonic biopsies mounted in Ussing chambers from both study populations showed an increase, p < 0.05, in both paracellular and transcellular permeability (assessed by FITC-dextran and HRP-flux, respectively) after stimulation with C48/80 when compared to vehicle (Figs 1 and 2), after 90 min.

Biopsies pre-stimulated for 20 min with β-glucan (0.5 mg/ml) and C48/80 showed an attenuation of the C48/80-induced paracellular hyperpermeability in elderly with GI symptoms, p < 0.01, but not in healthy controls (Fig. 1). Pre-stimulation with β-glucan (0.5 mg/ml) only gave a increase in paracellular permeability, p < 0.05, compared to vehicle in the elderly with GI symptoms but no significant difference could be observed in the healthy controls (Fig. 1).

A similar pattern was seen for transcellular permeability, where pre-stimulation with β-glucan (0.5 mg/ml) and C48/80 showed an attenuation, p < 0.001, in the induced transcellular permeability in elderly with GI symptoms (Fig. 2). Likewise, pre-stimulation with the β-glucan failed to display any significant attenuating effect in the healthy controls (Fig. 2b). Pre-incubation with β-glucan (0.5 mg/ml) only showed an increase in transcellular permeability, p < 0.01, compared to vehicle in the elderly with GI symptoms but not healthy controls (Fig. 2). Due to technical problems the following number of participants had to be excluded from each respective analysis; elderly with GI symptoms (FITC; n = 1, HRP; n = 2).

### Arabinoxylan attenuates MC-induced hyperpermeability in healthy controls but only transcellular permeability in elderly with GI symptoms

**Figure 3:**
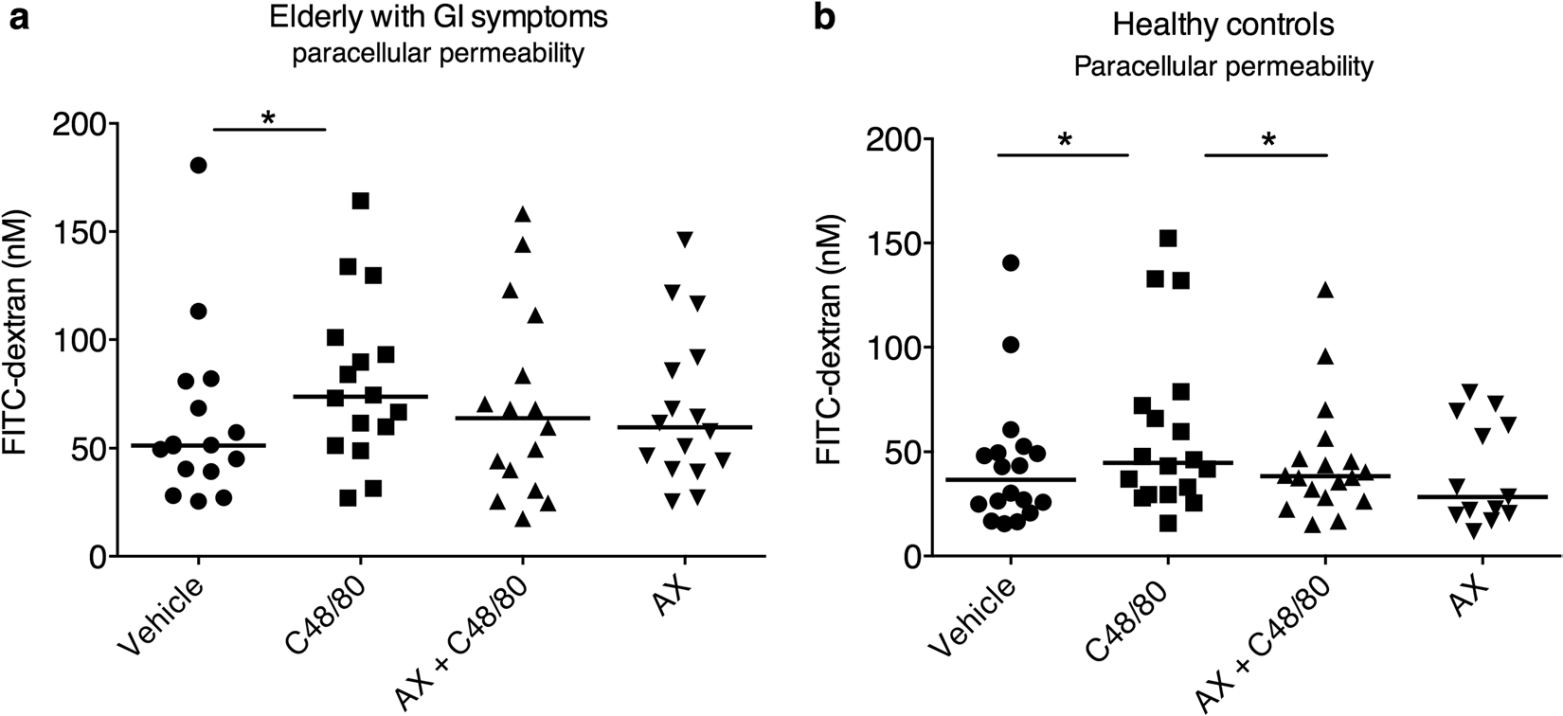
Effects of the wheat-derived arabinoxylan (AX) on colonic paracellular permeability in biopsies mounted in Ussing chambers. Stimulation with Compound (C) 48/80 (10 ng/ml) resulted in a significantly higher paracellular permeability compared to vehicle (**a**,**b**). Co-stimulation with AX (0.1 mg/ml) showed a significant decrease of C48/80-induced hyperpermeability on paracellular passage in only the healthy controls (**b**). Stimulation with AX only had no significant effect on neither paracellular nor transcellular permeability compared to vehicle. *p < 0.05, **p < 0.01, ***p < 0.001, ns = non-significant. Two elderly with GI symptoms and 3 healthy controls had to be excluded from the FITC-analysis due to technical problems, hence elderly with GI symptoms; n = 16 and healthy controls; n = 18 (AX only, n = 13).

**Figure 4:**
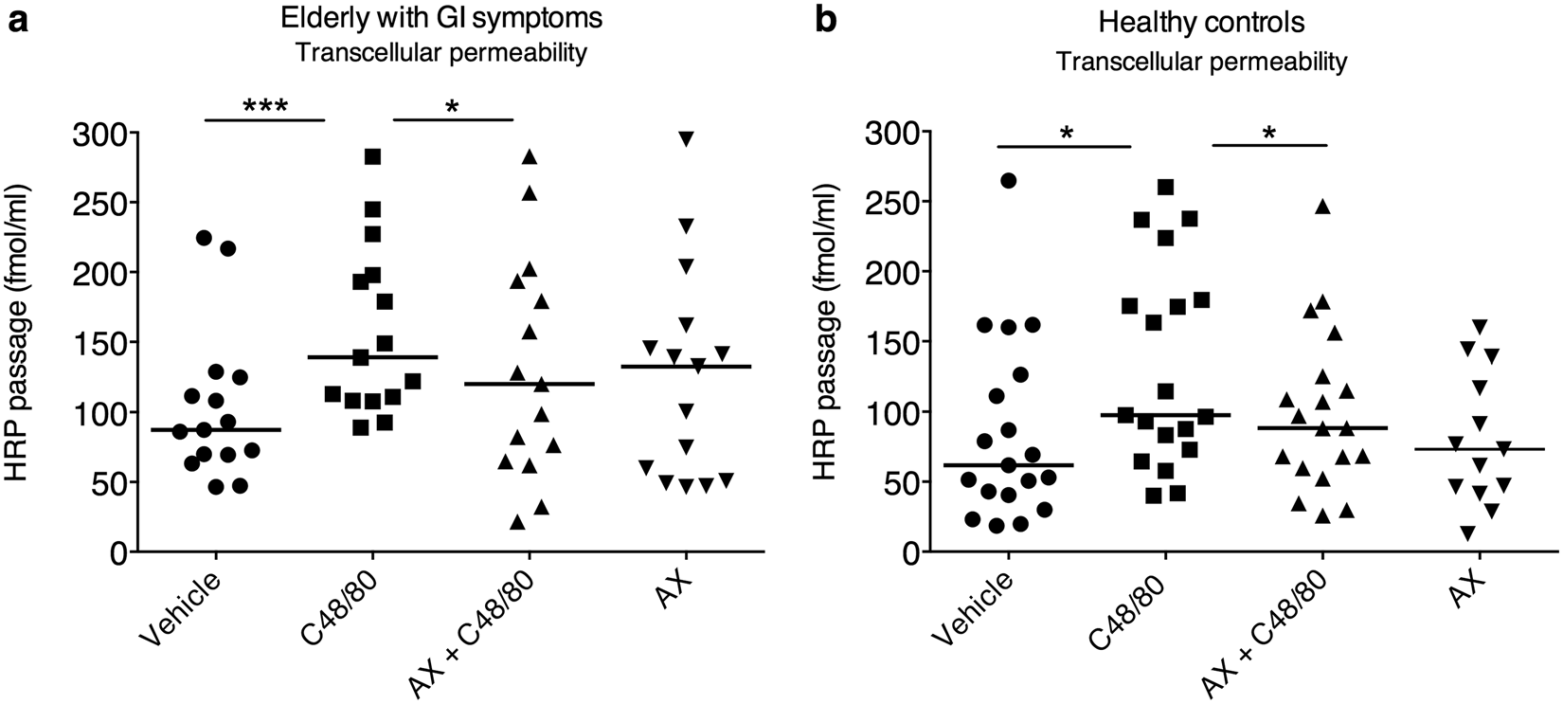
Effects of the wheat-derived arabinoxylan (AX) on colonic transcellular permeability in biopsies mounted in Ussing chambers. Stimulation with Compound (C) 48/80 (10 ng/ml) resulted in a significantly higher transcellular passage of horseradish peroxidase (HRP) compared to vehicle (**a**,**b**). Co-stimulation with AX (0.1 mg/ml) showed a significant decrease of C48/80-induced transcellular permeability in both elderly with GI symptoms and healthy controls (**b**). Stimulation with AX only had no significant effect on neither paracellular nor transcellular permeability compared to vehicle. *p < 0.05, **p < 0.01, ***p < 0.001, ns = non-significant. Three elderly with gastrointestinal (GI) symptoms and 2 healthy controls had to be excluded from the FITC-analysis due to technical problems, hence elderly with GI symptoms; n = 15 and healthy controls; n = 19 (AX only, n = 13).

A significant increase in para – and transcellular permeability was observed after stimulation with C48/80 compared to vehicle for both elderly individuals suffering from diarrhoea and/or constipation, p < 0.05, and healthy controls, p < 0.05. Pre-stimulation for 20 min with arabinoxylan (0.1 mg/ml) only attenuated C48/80 induced transcellular hyperpermeability in elderly individuals, p < 0.05, while a reduction in both paracellular and transcellular hyperpermeability was observed after 20 min pre-stimulation with arabinoxylan across colonic biopsies from healthy individuals, p < 0.05. Pre-stimulation with only arabinoxylan did not generate any changes compared to vehicle in any of the groups. Figures 3 and 4 illustrate the Ussing chamber results for both elderly with GI symptoms and healthy controls on paracellular and transcellular permeability, respectively. Due to technical problems the following number of participants had to be excluded from each respective analysis; elderly with GI symptoms (FITC; n = 2, HRP; n = 3), controls (FITC; n = 3, HRP; n = 2).

### Elderly with GI symptoms display higher basal permeability compared to healthy controls

**Figure 5:**
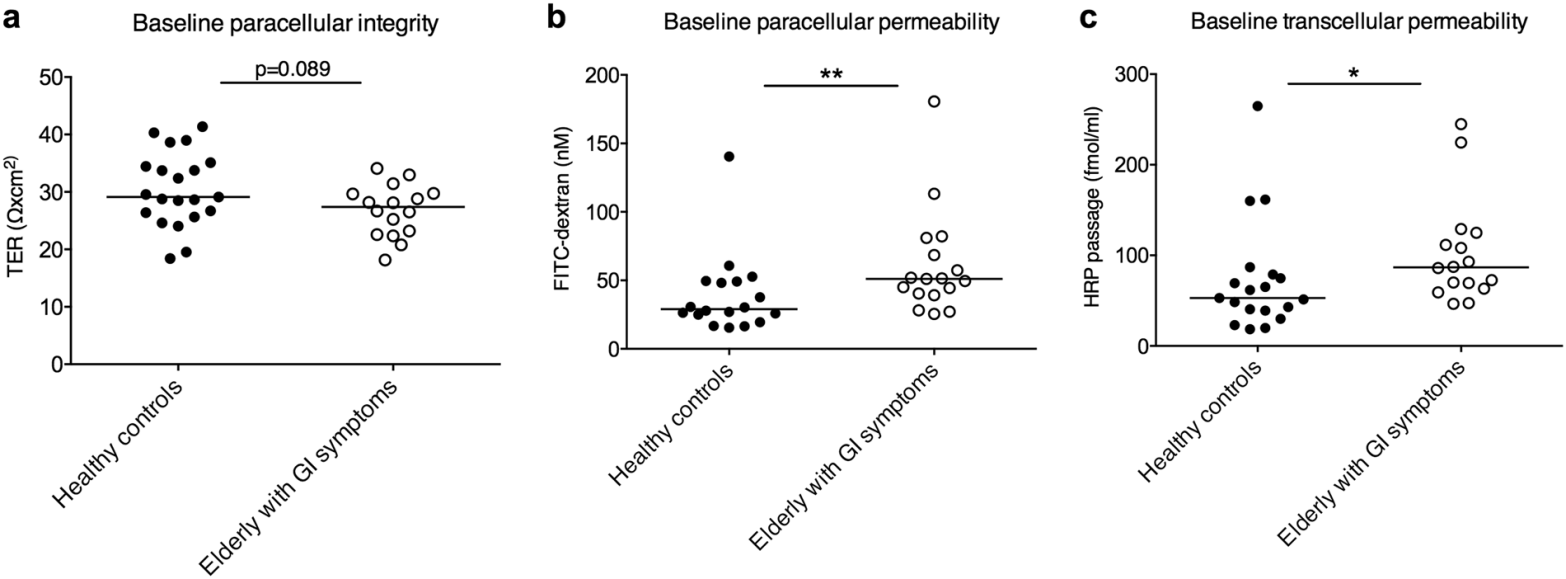
Baseline levels of paracellular integrity and permeability in the 2 study populations. (**a**) Paracellular integrity as measured by transepithelial resistance (TER) in the Ussing chambers displayed a close to significantly (p = 0.089) lower paracellular integrity in the group of elderly with gastrointestinal (GI) symptoms (n = 16) compared to healthy controls (n = 21). (**b**) Paracellular permeability as measured by FITC-dextran flux showed significantly higher permeability in elderly with GI symptoms (n = 17) compared to healthy controls (n = 18). (**c**) Transcellular permeability as measured by horseradish peroxidase (HRP) flux displayed a significantly higher permeability in elderly with GI symptoms (n = 16) compared to healthy controls (n = 19). Data (0 min) is presented as a line intersecting the median and each dot represents one participant, *p < 0.05, **p < 0.01. Data from one older adult was excluded from the FITC analysis and two elderly from the TER and HRP analysis due to technical problems, therefore the number of elderly for FITC – and HRP results were 17 and 16, respectively. Data from three healthy controls were excluded from the FITC analysis and two healthy controls from the HRP analysis due to technical problems, therefore the number of healthy controls for FITC – and HRP results were 18 and 19, respectively.

Comparing TER, the electrophysiological parameter for paracellular integrity, after equilibration (before the administration of C48/80, NPS and permeability markers), between the different study populations revealed that elderly with GI symptoms had a close to significantly lower TER than the healthy controls, p = 0.089 (Fig. 5a). The paracellular marker FITC-dextran showed a significant difference, p < 0.01, of elderly with GI symptoms having two times higher FITC-dextran flux compared to healthy controls (Fig. 5b). The transcellular marker HRP showed a significant ≈ 60% higher transcellular permeability within the same group compared to the healthy controls, p < 0.05 (Fig. 5c). Due to technical problems the following number of participants had to be excluded from each respective analysis; controls (FITC; n = 3, HRP; n = 2), elderly with GI symptoms (FITC; n = 1, TER and HRP; n = 2).

### No difference in response to C48/80 and number of responders between the study populations

The C48/80 induced approximately 2 times higher para – and transcellular permeability compared to vehicle in both study populations (Figs 1 and 2). All data were normalised to vehicle and the fold change in hyperpermeability was compared between the two study groups (Supplementary Fig. S2). No significant difference could be observed between the two populations in C48/80 induced para – and transcellular permeability. Data from elderly with GI symptoms was also stratified based on subjects with constipation/diarrhoea or both but no significant differences could be detected. Also, no significant difference could be observed in the number of responders to C48/80 between the healthy controls and elderly with GI symptoms, using Fishers exact test (Supplementary Table S4). Similar stratification based on constipation/diarrhoea or both was performed but showed no significant differences. Due to technical problems the following number of participants had to be excluded from each respective analysis; controls (FITC; n = 3, HRP; n = 2), elderly with GI symptoms (FITC; n = 1, HRP; n = 2).

### No correlation between baseline – and C48/80 induced permeability against GI symptoms or psychological distress in elderly with GI symptoms

The elderly with GI symptoms did not display any significant correlation between baseline permeability of either FITC-dextran or HRP flux against scores on diarrhoea/constipation and the subdomains of the Hospital Anxiety and Depression Scale (HADS).

No significant correlation was found between an increase in permeability caused by C48/80 and the questionnaire scores (Table 3). Data from elderly with GI symptoms was further stratified based on subjects experiencing either constipation and/or diarrhoea but no significant differences were detected. Moreover, no significant correlations, neither positive nor negative, were observed between baseline permeability, C48/80 induced permeability+/− dietary fibre and GI symptoms, psychological distress, BMI and fibre intake (Supplementary Tables S5 and S6). Moreover, the results were not affected by confounding factors (i.e. medication and smoking) as assessed by stratification.

**Table 3:** Spearman correlation coefficients (*r*) shown between questionnaires scores on gastrointestinal (GI) symptoms and psychological distress against baseline – and C48/80 induced permeability in older adults with GI symptoms.

	**Median (IQR)**	Baseline permeability				Compound (C) 48/80 induced permeability (fold change)			
		*Paracellular permeability n* = 17		*Transcellular permeability n* = 16		*Paracellular permeability n* = 17		*Transcellular permeability n* = 16	
		***r***	***p***	***r***	***p***	***r***	***p***	***r***	***p***
GSRS score									
*Diarrhoea*	1.7 (1.0–2.2)	−0.04861	0.8533	0.2543	0.3401	0.4221	0.0924	0.1279	0.6368
*Constipation*	3.3 (1.9–4.3)	0.1564	0.5461	0.4124	0.1131	−0.01355	0.9602	0.03548	0.8962
HADS score									
*Anxiety*	5.0 (0.5–6.5)	0.0267	0.9297	0.2691	0.3712	−0.03778	0.8994	−0.02774	0.9283
*Depression*	2.0 (0–4.0)	0.2487	0.3878	0.3667	0.2164	−0.2577	0.3703	0.02558	0.9339
*HADS total score*	5.0 (1.5–10.0)	0.1173	0.6882	0.2739	0.3627	−0.07744	0.7923	0.06639	0.8294

GSRS score grading. HADS score grading.

GSRS – Gastrointestinal Symptoms Rating Scale, moderate = 2–4 points. HADS – Hospital Anxiety and Depression Scale, normal = 0–7 points, mild = 8–10 points.

## Discussion

The present study focused particularly on elucidating the effect of NPS in attenuating stress-induced colonic intestinal barrier function in elderly suffering from GI symptoms. By performing experiments *ex vivo* using the Ussing chamber technology we found that both yeast-derived β-glucan and arabinoxylan were able to attenuate MC-induced transcellular permeability in elderly suffering from GI symptoms compared to healthy individuals. However, only β-glucan was found to reduce MC-induced paracellular permeability in elderly with GI symptoms.

Elderly reporting GI symptoms displayed increased baseline permeability compared to healthy controls, however, this did not affect the ability of C48/80 to induce colonic hyperpermeability. Half of the elderly individuals were found to take medications in relation to cardiovascular disorders. Two of the participants were prescribed acetylic acid (ASA, 75 mg) towards hypertension. ASA is known to affect the intestinal barrier negatively and may induce increased permeability^29^. However intake of ASA or any other medication was found not to affect the intestinal permeability. This is in accordance with a recent study from our group demonstrating that the use of pharmaceutical agents did not affect plasma levels of zonulin, an indirect marker of small intestinal permeability^24^.

The observed elevated baseline permeability among elderly with GI symptoms is in line with previous results where we, among others, have identified an increase in small bowel permeability among elderly^30^ and those suffering from GI symptoms^24^. A recent study further shows that aged mice display an increased colonic permeability compared to young mice which trigger systemic inflammation, due to age-associated changes of the gut microbiota^31^. Previous studies have shown that diet-driven alterations of the gut microbiota are associated with a decline in health upon ageing^32^. Hence it is possible that elderly individuals suffering from GI symptoms display an altered gut microbiota compared to healthy controls, potentially inclined by a change in dietary pattern, that contribute to an elevated colonic permeability.

Interestingly, we did not observe any change in basal colonic permeability between elderly with no GI symptoms and young adults as demonstrated by our findings from the multi sugar test. These results are further supported by preliminary data showing no influence of age on intestinal permeability when investigating surgical tissue from adult and old individuals suffering from cancer in Ussing chambers. Thus, our results indicate that the diminished intestinal barrier function in elderly suffering from GI symptoms is merely due to the symptoms and not to age.

It is possible that the alterations of microbiota composition between elderly with no GI symptoms and healthy young adults in the present study are too modest to influence colonic permeability. This could be due to geographical location as both elderly and adult individuals in Sweden have been found to have a high abundance compared to other European countries of the intestinal barrier promoting bacterium *Faecalibacterium prausnitzii*^33–35^.

Moreover, the elderly participants in the present study is a small heterogenous study group where many factors except age influence the composition of the gut microbiota making it difficult to identify specific changes only associated with age that correlate to colonic permeability. Nevertheless, the lack of analyses of the gut microbiota composition and correlation to the colonic permeability is a limitation of the study. It should also be noted that assessment of the intestinal barrier function of elderly with no GI symptoms using the Ussing chamber technique would have been preferable, as this technique makes it possible to thoroughly investigate the colonic barrier function. However, this population was particularly difficult to recruit as the methodology is invasive and requires a sigmoidoscopy for collection of colonic biopsies.

The severity of GI symptoms or psychological distress was further not found to be associated with increased baseline permeability. This could be due to the relatively low number of participants in the study and experience of moderate symptoms only as no participant did report a severe symptom. In addition, a recent study by Bednarska *et al*. found no correlation between symptom severity, colonic permeability and the number of MCs in IBS patients^19^. Hence, indicating that GI symptoms might not be associated with an impaired intestinal barrier *per se* but instead the intestinal epithelium might be less stress resilient in elderly suffering from GI symptoms.

The differential effect of the two NPS on the colonic barrier function might be due to specific changes in the level and/or distribution of immune cells related to the symptoms of constipation and/or diarrhoea. Previous studies suggest an increased number of MCs in individuals suffering from constipation and diarrhoea^36–38^, which might be one of the reasons behind the increased baseline permeability observed in the present study. In addition CRH is known to be associated with psychological distress, conditions that are common among the elderly and that we recently identified as associated with GI symptoms and an increased intestinal permeability^24^. In addition, macrophages have been found to have an important role in taking up yeast-derived β-glucans and processing them into compounds with higher bioactive capabilities^39^. Hence, a potentially higher number of macrophages in the intestine compared to healthy controls could potentially contribute to the observed differences in response towards MC-induced hyperpermeability. Therefore, it is a limitation that the presence and distribution of immune cells were not investigated in the present study. It is also important to consider when interpreting the results that females are overrepresented in our elderly study population (18:2), which have been observed in previous studies^24,40^. Hence, suggesting that the effects observed of β-glucan is mainly applicable to elderly females. The high proportion of females in the study population might reflect that elderly females are more prone to suffer from GI symptoms than men^40,41^, but could also be due to a larger interest among women to participate in research studies. Hence, other recruitment procedures might be needed in order to attract men to participate. This is important to consider when recruiting elderly study participants.

Interestingly, β-glucan but not arabinoxylan induced a significantly higher para – and transcellular permeability compared to vehicle in elderly with GI symptoms. However, this effect was not reflected in the TER values. The increased intestinal permeability induced by β-glucan could be due to contamination of lipopolysaccharide (LPS) in the purification process of β-glucan from *Saccharomyces cerevisiae*. However, endotoxin tests performed by the manufacturer revealed no contamination of LPS. Previous studies have shown that β-glucan can bind to receptors on phagocytic cells promoting an oxidative burst of reactive oxygen species (ROS)^42^. This could potentially induce an increased intestinal permeability^43–45^. However, increased ROS levels are also important signalling molecules in the body to maintain physiological functions^46^ such as gut barrier homeostasis^47^. Hence, it would have been important to assess the levels and composition of ROS potentially induced by β-glucan in the present study to further understand the mechanisms behind the β-glucan induced intestinal permeability. In addition, it might be possible that yeast-β-glucan initiates a cascade of immune responses that lead to a transient hyperpermeability in elderly with GI symptoms, likely due to a less stress-resilient barrier compared to healthy adults. However, no significant correlations could be found between the β-glucan induced hyperpermeability and age, BMI, GI symptoms, HADS and fibre intake. In future studies it will be important to further elucidate the dual role of β-glucan on the intestinal barrier function. Thorough mechanistic studies are needed in order to understand how β-glucan can attenuate MC-induced hyperpermeability while simultaneously induce an increased intestinal permeability in elderly. Interestingly, the number of participants responding to C48/80 was similar among the elderly with GI symptoms and healthy controls. Moreover, there was no difference in the level of hyperpermeability between the two groups.

Thus, indicating that GI symptoms in elderly do not induce a change in the level of MC. Hence, further demonstrating that arabinoxylan and β-glucan exert their effect through different mechanisms of action.

Arabinoxylan was further found to only attenuate C48/80-induced transcellular hyperpermeability in elderly with GI symptoms. This is in line with previous animal data suggesting that arabinoxylan has a more pronounced effect on transcellular permeability^48^ through TJ-independent mechanisms. This effect could be of clinical relevance as elderly with diarrhoea are commonly affected by enteric infections caused by bacteria invading through the transcellular route^18,49^.

The outlined study only investigates the direct effect of NPS on the intestinal mucosa *ex vivo* and did not investigate changes after dietary supplementation. The effect of arabinoxylan might be predominantly mediated by promoting the growth of butyrate producing bacteria in the intestine through fermentation, generating many beneficial effects on the intestinal barrier function^50,51^. Therefore, clinical trials need to be performed in order to thoroughly elucidate the effect of arabinoxylan and yeast-derived β-glucan on the intestinal barrier function among elderly. This is particularly important as ageing is associated with a change in microbiota composition which could influence the intestinal permeability^31,52^.

In conclusion our results demonstrate that NPS affect the intestinal epithelium differently by promoting either transcellular and/or paracellular permeability. We showed that yeast-derived β-glucan and arabinoxylan affect transcellular permeability but only β-glucan reduced paracellular permeability in response to C48/80. These novel findings indicate that dietary supplementation exert differential effects on the colonic barrier function and could be important in the treatment of moderate constipation and/or diarrhoea in elderly.

## Methods and Material

### Subjects for Ussing experiments

Twenty elderly individuals with GI symptoms were recruited for Ussing experiments, based on a score ≥3 on the Gastrointestinal Symptoms Rating Scale (GSRS) for diarrhoea and/or constipation. The distribution of GI symptoms in the elderly cohort was as follows: constipation (68%), diarrhoea (21%) and mix (11%). Two elderly participants were excluded based on violation of the inclusion/exclusion criteria (remaining n = 18). In addition, a healthy population consisting of 23 subjects (age ≥18 years) was recruited through advertisements in local and regional newspapers as well as through posters at the University and meeting points for elderly. Two participants were excluded based on violation of inclusion/exclusion criteria (remaining n = 21). Supplementary Table S2 lists the inclusion/exclusion criteria for the both study populations and Supplementary Fig. S1 illustrate a flow chart over the study. Demographic data and information about allergies and active intake of MC stabilisers was recorded in the CRF of all study participants. None of the study participants were actively taking mast cell stabilisers at the time of participation. All demographic data over gender distribution, age, BMI and medications can be viewed in Table 1. Dietary intake was estimated through FFQ completed at study start. The FFQ has previously been validated in a Swedish population^53^ and consists of 66 categories of food that evaluates the dietary pattern over a year.

### Ethical consideration

The Regional Ethics Committee in Uppsala, Sweden approved the study (dnr 2013/037 and 2015/357). The study followed the principles of the Helsinki declaration with all participants having signed the informed consent before starting the study.

### Evaluation of GI symptoms and psychological distress

#### Gastrointestinal Symptoms Rating Scale (GSRS)

The questionnaire GSRS was used to evaluate the GI symptoms of the elderly before entering the study. The GSRS reliability and validity is well documented^54^. The scale includes 5 symptoms (e.g. reflux, abdominal pain, indigestion, diarrhoea and constipation), assessed with 15 items, scored from 1 to 7 depending on their severity. A score of 1 represents “no problems” and score 7 represents “severe problems”. The severity of symptoms may be graded as no problems (1 point), mild (1–2 points), moderate (2–4 points), and severe (4–7 points).

#### The Hospital anxiety and depression scale (HADS)

HADS is an extensively used validated instrument for the evaluation of psychological distress in medical settings, as well as in elderly^55,56^. The instrument consists of 14 items, consisting of two subscales for assessment of anxiety or depression. The total score is used as a measure of psychological distress.

### Non-invasive multi-sugar test for investigating the influence of age on colonic permeability

To compare baseline intestinal permeability between elderly with no GI symptoms and young healthy adults a non-invasive multi-sugar test was performed. Study participants (≥65 years) with no GI symptoms (n = 31) and young adults (n = 17) were recruited through advertisements in local and regional newspapers as well as through posters placed at pin boards at the University and at specific meeting points for elderly. The young healthy control group was comprised of young adults where 2 participants did also participate in the Ussing chamber experiments. All demographic data over gender distribution; age, BMI and medications and GI symptoms can be viewed in Supplementary Table S1. Fasted study participants were instructed to drink a multi-sugar solution containing five sugars; 1 g Sucrose (Nordic Sugar, Sweden), 1 g Lactulose (Solactis, France), 0,5 g L-rhamnose (BioGaia, Sweden), 1 g Sucralose (Univar, Sweden) and 1 g Erythritol (Ingredi, Sweden) dissolved in 150 ml of tap water. Urinary output collected between 5–24 h reflected the colonic permeability. The sucralose to erythritol ratio (S/E) was particularly analysed in the present study to assess colonic permeability of elderly with no GI symptoms and young adults. Blood samples were collected prior to study start for assessment of creatinine as a measurement of renal function. A detailed description of the multi-sugar test can be found in the supplementary methods section.

### Ussing chamber experiment

#### Collection of colonic biopsies for Ussing chamber experiments

The participants did not undergo any bowel cleansing procedure (but did fast overnight prior to the appointment) in order to avoid interference with the mucosa. Twelve biopsies were taken with a biopsy forceps without a central lance from sigmoid colon^55^ and were immediately put in 4 °C oxygenated modified transport Krebs-Ringer bicarbonate buffer (KRB; 115 mM NaCl, 1.25 mM CaCl_2_, 1.2 mM MgCl_2_, 2 mM KH_2_PO_4_, and 25 mM NaHCO_3_, pH 7.35) and transported to the laboratory within 10 minutes.

#### Experimental set up

Each chamber held one biopsy with each intervention run in duplicates. Two unstimulated biopsies were used as controls (vehicle) while two biopsies were stimulated only with C48/80 (Sigma Chemical Co, MO, USA), a well-documented MC degranulation mediator that resembles CRH by inducing increased paracellular and transcellular permeability^17,28^. Two biopsies were stimulated with C48/80 + β-glucan (0.5 mg/ml), two with C48/80 + arabinoxylan (0.1 mg/ml) and two with β-glucan (0.5 mg/ml)/arabinoxylan (0.1 mg/ml) only.

#### Ussing chamber experimental procedure

Colonic biopsies from 20 elderly with diarrhoea/constipation and 23 healthy controls were mounted in the Ussing chambers (Harvard apparatus Inc., Holliston, MA, USA), as described previously^57,58^. Soluble β-1,3/1,6-glucan (0.5 mg/ml) from Baker’s yeast (Biothera, Eagan, MN, USA) or arabinoxylan (0.1 mg/ml) (Bioactor BV, the Netherlands and Nofima, Norway) was added to the mucosal side of designated chambers, and after 20 min, C48/80 (10 ng/ml) was added to the serosal side. Due to a limited number of chambers available for experiment, biopsies from a total of 13 healthy controls were used for the β-glucan experiments. The concentration of arabinoxylan was based on experiments evaluating the concentrations 0.1 – and 0.05 mg/ml, with the former showing most promising effects and subsequently used as the final concentration. The concentrations of C48/80 and soluble β-1,3/1,6-glucan were based on previous experiments^28^. The paracellular marker FITC-dextran 4000 (Sigma) and the 45 kD transcellular marker HRP (Type VI; Sigma) were added to the mucosal sides (2.5 nM and 10^−5^ M, respectively). Serosal samples were collected at 0 and 90 min. The Isc, TER and PD were monitored throughout the experiments to ensure good tissue viability. To ensure viability by the end of the experiment, cAMP-dependent Cl secretagogue, forskolin (10^−5^ M) (Sigma), was added to both sides of the biopsies and ∆Isc was recorded as a measure of tissue viability. Biopsies that did not react to forskolin through changes in Isc, or had a PD >+0.5 mV^55^, were judged not suitable for inclusion in subsequent analysis.

#### Measurement of FITC-dextran 4000 and HRP

FITC-dextran passage was measured at λ_ex_ = 485 nm and λ_em_ = 530 nm using EnSpire® Multimode Plate Reader (Perkin Elmer, MA, USA). HRP passage was measured with QuantaBlu™ Fluorgenic Peroxidase Subtrate Kit (Pierce, Rockford, USA) as previously described^29^. Results from the FITC-dextran and HRP-analyses were expressed as Δ90-0 min, and samples were measured in duplicates against a standard curve.

### Statistical analysis

The normality of the data was assessed using the Shapiro-Wilk test and by visualising the data in histograms. Non-parametric data are shown as median and presented as scatter plots. The pair-wise comparisons within same groups were performed using Wilcoxon matched-pairs signed rank test. Students’ t-test for paired comparisons was used on parametric data. Comparisons between the two study groups were done using Mann-Whitney U test. All subjects with a <20% increase in permeability compared to unstimulated biopsies (vehicle) were considered non-responders to C48/80. Fishers exact test was used to test differences among responder’s vs. non-responders to C48/80 between the two study populations. Baseline, C48/80-induced –and β-glucan stimulated permeability was correlated against the diarrhoea - and constipation scores on the GSRS domains, in addition to anxiety, depression, total HADS score, age, BMI and fibre intake, using Spearman correlation analysis and correcting for multiplicity using Bonferroni correction. To investigate the influence of confounding factors we made a stratified analysis for each factor (Cardiovascular drugs, Gut regulating substances and smoking) to verify the stability of the results. Differences of p < 0.05 were considered significant.

## Electronic supplementary material

Supplementary information

## Data Availability

The datasets generated during and/or analysed during the current study are available from the corresponding author on reasonable request.
