# Supplementary material for: Differential effects of dietary fibres on colonic barrier function in elderly individuals with gastrointestinal symptoms
Source: Sci Rep. 2018 Sep 7;8:13404. doi: 10.1038/s41598-018-31492-5 (PMC6128877; doi:10.1038/s41598-018-31492-5)
Supplement: Supplementary file 1 — Supplementary information [file 41598_2018_31492_MOESM1_ESM.pdf]

Differential effects of dietary fibres on colonic barrier function in elderly individuals with gastrointestinal symptoms

JP Ganda Mall[1,2], L Löfvendahl[1], CM Lindqvist[1], RJ Brummer[1], ÅV Keita[3], I Schoultz [2*]

[1]Nutrition-Gut-Brain Interactions Research Centre and [2]Nutrition and physical activity research centre, School of Medical Sciences, Faculty of Medicine and Health,
Örebro University, Örebro, Sweden
[3]Department of Clinical and Experimental Medicine, Linköping University, Linköping, Sweden.

Corresponding Author: Dr Ida Schoultz, Örebro University, 703 62 Örebro, Sweden, +46 (0) 19 303209, ida.schoultz@oru.se

SUPPLEMENTARY METHODS, TABLES AND FIGURES

Supplementary method text

In vivo gastrointestinal permeability measured by a non-invasive multi sugar test

A non-invasive multi-sugar test, quantifying 24h urinary excretion of five different ingested sugars was used to assess gut permeability. The multi sugar test allows for the assessment of gastroduodenal, ileal and colonic intestinal permeability. In the present study it was used particularly for assessment of colonic permeability. Fasted study participants were instructed to drink a multi-sugar solution containing five sugars; 1 g Sucrose (Nordic Sugar, Sweden), 1 g Lactulose (Solactis, France), 0,5 g L-rhamnose (BioGaia, Sweden), 1 g Sucralose (Univar, Sweden) and 1 g Erythritol (Ingredi, Sweden) dissolved in 150 ml of tap water. The urinary output between 5-24h was used and analysed in the present study for assessment of colonic permeability. The participants were not allowed to ingest any foods or drinks except for water throughout the first 5 hours. During collection of urinary output between 5-24h the participants were allowed to drink and return to their habitual dietary pattern, with the exception of caffeine-based products, alcohol, spicy food, drinks or sweets containing the same sugars as the multi-sugar mix. The sucralose to erythritol (S/E) ratio in the 5-24h urinary output represented colonic permeability. After collection of the 24h urinary output, the total volume was quantified and urine aliqouts were prepared by the participants using a built-in vacuum system in the collection jar (Sarstedt, Sweden) and stored at -20°C. Samples were transferred to the lab within one week and stored at -20°C until further analysis. Urinary sugars were analysed using liquid chromatography-mass spectrometry (LC-MS) at Örebro University, Sweden.

Measurement of sugar probes in urine samples

Sample preparation and analysis

1 ml of urine was centrifuged at 21 000 RCF for 25 minutes at a temperature of 4°C. The supernatant was collected and 50 µL was transferred to liquid chromatography vials. The urine aliquots were diluted to a volume of 1 mL with a composition of 80:20 acetonitrile:water. Before analysis, all samples were vortexed for 10 seconds and centrifuged for 15 min at 8000 RCF. Analysis was performed on an Acquity UPLC coupled to a Quattro Premier XE UPLC–MS/MS system (Waters Corporation, Milford, USA) with an atmospheric electrospray interface operating in negative ion mode. Analytes were separated on an Acquity BEH Amide column (1.7 µm, 2.1 x 100 mm) (Waters Corporation, Milford, USA). Column temperature was 50°C, injection volume 10 µl, flow rate 0.17 mL/min. An isocratic method was used with a mobile phase of 0.1 % NH_4OH in acetonitrile and water (70:30). Blanks and external standards were frequently injected during the analysis to control for carry over and monitor instrument stability. Mass-analysis was performed in the multiple reaction monitoring (MRM) mode by monitoring two product ions for each sugar analysed (341.16>100.80 and 341.16>160.80 for lactulose, 163.20>59.00 and 163.20>103.00 for rhamnose). Sucralose and erythriol, were quantified using 3-point standard addition curves. An inhouse control sample was included in each batch.

Supplementary Table S1. Demographic data showcasing the baseline characteristics between elderly with no gastrointestinal symptoms and healthy adults undergoing assessment of colonic permeability using the non-invasive multi-sugar test.

	Elderly with no gastrointestinal symptoms	Healthy adults	P value
Gender	**n=31**	**n=17**	
Female, n (%)	11 (35%)	13 (76%)	
Male, n (%)	20 (65%)	4 (24%)	
Age, mean ± std	70±4.6	26±3.7	P<0.001
BMI, median (IQR)[1]	25 (22.0-27.0)	22 (19.0-23.0)	P<0.001
Smokers, n (%)	0 (0%)	0 (0%)	
**Gastrointestinal symptoms rating scale, median (IQR)**			
Diarrhoea	1 (1.0-1.7)	1.3 (1.0-1.3)	ns
Constipation	1 (1.0-1.3)	1.2 (1.0-1.6)	ns
Total	1.3 (1.2-1.6)	1.4 (1.1-1.9)	ns
**Medications**	**%**	**%**	
Cardiovascular drugs[2]	45.2	0	
Gut regulating drugs[3]	9.7	0	
NSAIDs[4]	0	0	
Others[5]	35.5	8.7	
Polypharmacy[6]	9.7	0	

[1]IQR – Interquartile range
[2]Cardiovascular drugs: antihypertensive medications, anti-coagulants, statins
[3]Gut regulating drugs: probiotics, fibres, laxatives, proton pump inhibitors
[4]NSAIDs – Non-steroid anti-inflammatory drugs
[5]Others – Thyroid drugs, sleeping pills, cough medicine, hormones, anti-depressant, contraceptives
[6]Polypharmacy – 5 or more drugs

Supplementary Table S2: Inclusion and exclusion criteria's for participation in the study from the 2 different study populations.

	**Inclusion criteria**	**Exclusion criteria**
Older adults with gastrointestinal symptoms	Informed consent signed by study participant	Gastrointestinal disease with strictures. malignancies and ischemia
	Age ≥ 55 years	Inflammatory bowel diseases
	Scoring above 3 on the dimensions for diarrhoea and constipation on the GSRS	Participation in other clinical trials in the past three months
	Mentally and physically fit to complete questionnaires during the study period	Intake of medications know to change the inflammatory status (i.e. proton pump inhibitors. antibiotic. anti-inflammatory medication (including NSAIDs)
Healthy subjects	Age ≥ 18 years	Previous abdominal surgery
	Informed consent signed by the study participant	A hypertonic condition demanding medical treatment
	Mentally and physically fit to complete questionnaires during the study period	Diagnosed psychiatric disease
		Lactose intolerance
		Usage of medical prescribed medications. expect oral contraceptives. during the 14 days preceding study start
		Premenstrual syndrome
		Pregnant or breast feeding
		Gastrointestinal disease with strictures. malignance's and ischemia
		Inflammatory bowel diseases
		Participation in other clinical trials in the past three months

Supplementary Table S3: Short circuit current (Isc) values (mean±SD) with 30 min intervals normalised to each participants respective 0 min value.

**Isc**	**0 min**	**30 min**	**60 min**	**90 min**	***Baseline corrected mean over time (30-90 min)***
Healthy controls (n=21)					
- Vehicle	100.0	99.9 ± 39.5	113.1 ± 46.6	123.7 ± 53.1	*112.2 ± 44.6*
- C48/80	100.0	103.8 ± 36.9	115.2 ± 48.9	127.1 ± 61.8	*115.3 ± 47.1*
- C48/80 + β-glucan *(n=13)*	100.0	115.4 ± 50.6	93.1 ± 55.0	124.0 ± 50.2	*110.8 ± 20.6*
- C48/80 + AX	100.0	93.7 ± 63.3	108.0 ± 74.2	129.0 ± 78.5	*110.1 ± 67.3*
- β-glucan *(n=13)*	100.0	107.7 ± 37.3	106.7 ± 17.7	103.5 ± 13.5	*106.0 ± 15.5*
- AX	100.0	100.0 ± 54.3	113.0 ± 75.5	104.0 ± 118.0	*107.9 ± 78.1*
Older adults with gastro-intestinal symptoms (n=16)					
- Vehicle	100.0	118.6 ± 47.5	129.9 ± 53.1	104.0 ± 24.2	*117.5 ± 21.1*
- C48/80	100.0	122.9 ± 47.7	116.7 ± 19.3	109.9 ± 28.8	*116.5 ± 17.9*
- C48/80 + β-glucan	100.0	129.6 ± 49.3	118.5 ± 48.9	109.0 ± 16.4	*119.0 ± 21.1*
- C48/80 + AX	100.0	124.1 ± 24.5	137.5 ± 27.8	131.8 ± 50.9	*131.2 ± 25.9*
- β-glucan	100.0	143.2 ± 64.2	109.8 ± 21.9	109.5 ± 12.6	*120.8 ± 26.4*
- AX	100.0	118.0 ± 48.0	134.9 ± 64.3	93.7 ± 168.7	*122.8 ± 75.1*
Arabinoxylan (AX). Compound 48/80 (C48/80)					

Supplementary Table S4: Distribution of responders versus non-responders against Compound (C) 48/80 mediated mast cell-induced hyperpermeability in both study populations.

	**Responders**	**Non-responders**
Older adults with gastrointestinal symptoms (n=18)	12	6
Healthy controls (n=21)	14	7
Fisher's exact test	Non-significant (p=1)	

Supplementary Table S5: Spearman correlation coefficients (*r*) shown between demographic parameters against baseline – and C48/80 induced permeability in older adults with GI symptoms.

	**Baseline permeability**				**Compound (C) 48/80 induced permeability**			
	Paracellular permeability n=17		*Transcellular permeability n=16*		*Paracellular permeability n=17*		*Transcellular permeability n=16*	
**Demographic parameters**	***r***	***p***	***r***	***p***	***r***	***p***	***r***	***p***
Age	-0.4174	0.0955	-0.5935	0.0171*	-0.3118	0.2218	-0.1365	0.6121
BMI	-0.02452	0.9256	-0.01619	0.9542	0.02452	0.9264	-0.09566	0.7234
% Fibre below RI[1]	-0.2328	0.3685	-0.2471	0.3550	-0.2206	0.3934	-0.3235	0.2213

RI[1] - recommended daily fibre intake according to the Nordic Nutrition Recommendations

*Significance was lost after Bonferroni correction

Supplementary Table S6: Spearman correlation coefficients (*r*) shown between demographic parameters against β-glucan induced permeability in older adults with GI symptoms.

	**Permeability after β-glucan stimulation**			
	Paracellular permeability n=17		*Transcellular permeability n=16*	
**Demographic parameters**	***r***	***p***	***r***	***p***
Age	-0.2062	0.4271	-0.3353	0.2042
BMI	-0.2036	0.4333	-0.1045	0.7002
% Fibre below RI[1]	-0.3995	0.1121	-0.4029	0.1217
**GSRS score**				
Diarrhoea	-0.05756	0.8263	0.2112	0.4324
Constipation	0.3732	0.1401	0.4494	0.0808
**HADS score**				
Anxiety	0.3511	0.2183	0.5437	0.0548
Depression	0.4589	0.0988	0.6083	0.0274*
HADS total score	0.3828	0.1768	0.5477	0.0527

RI[1] - recommended daily fibre intake according to the Nordic Nutrition Recommendations (25g/day)

*Significance was lost after Bonferroni correction

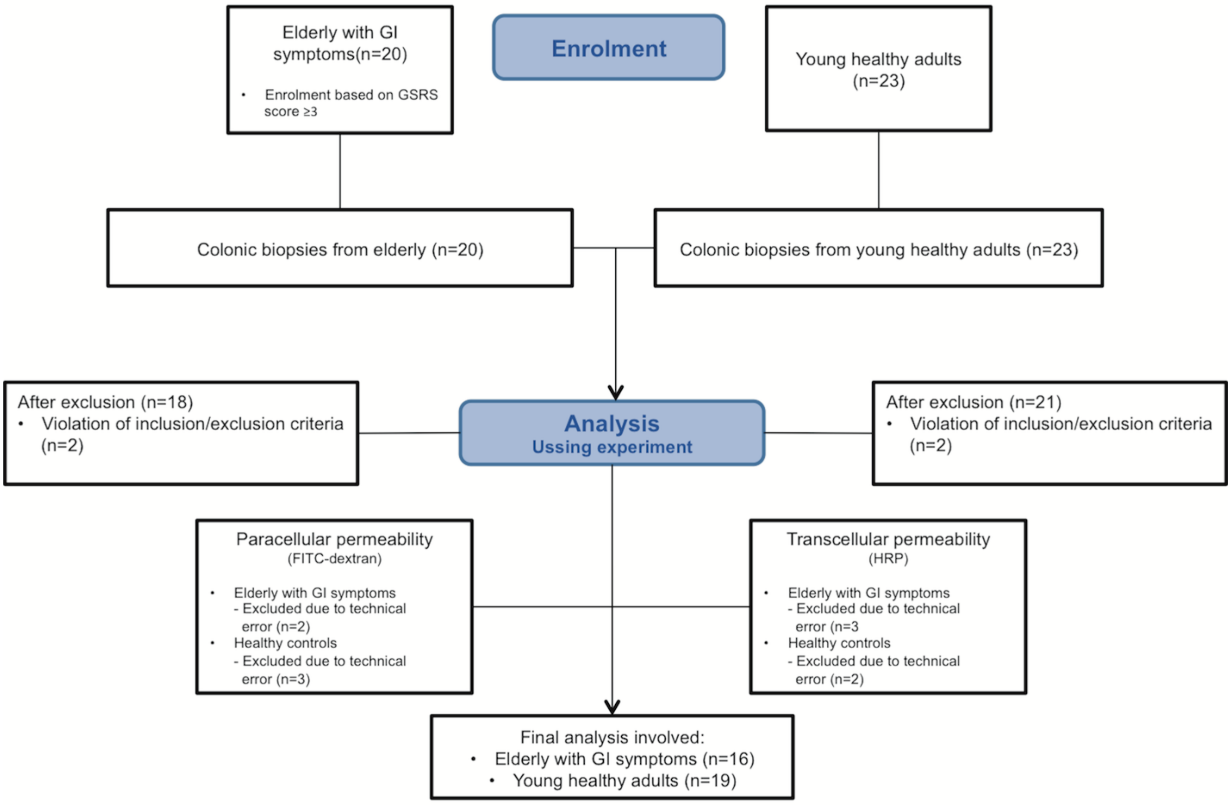

Supplementary Figure S1. Flow chart of all involved study participants from beginning to end of final analysis of permeability markers in the study.

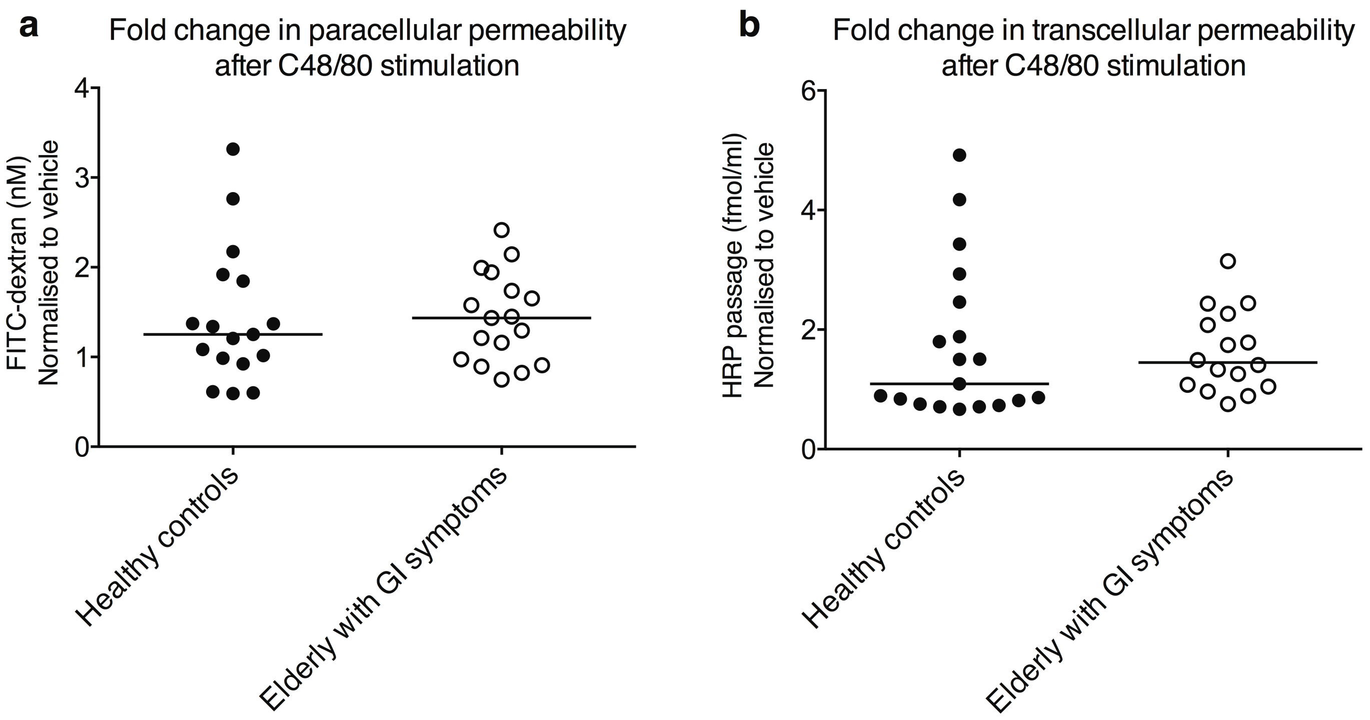

Supplementary Figure S2. Fold change in permeability after stimulation with Compound (C) 48/80 compared to vehicle. No significant differences could be detected for neither paracellular (a) nor transcellular permeability (b) upon C48/80 stimulation in neither elderly with gastrointestinal (GI) symptoms (FITC-dextran, n=17; horseradish peroxidase (HRP), n=16) nor healthy controls (FITC-dextran, n=17; HRP, n=19). Data (Δ90—0 min) is presented as a line intersecting the median and each dot represents one participant, ns=non-significant. Data from one older adult was excluded from the FITC analysis and two elderly from the HRP analysis due to technical problems, therefore the number of elderly for FITC – and HRP results were 17 and 16, respectively. Data from three healthy controls were excluded from the FITC analysis and two healthy controls from the HRP analysis due to technical problems, therefore the number of healthy controls for FITC – and HRP results were 18 and 19, respectively.
